# Promising non-model microbial cell factories obtained by genome reduction

**DOI:** 10.3389/fbioe.2024.1427248

**Published:** 2024-08-05

**Authors:** Giulia Ravagnan, Jochen Schmid

**Affiliations:** Institute of Molecular Microbiology and Biotechnology, University of Münster, Münster, Germany

**Keywords:** genome reduction, chassis, genetic engineering, non-model microbes, bacteria

## Abstract

The development of sustainable processes is the most important basis to realize the shift from the fossil-fuel based industry to bio-based production. Non-model microbes represent a great resource due to their advantageous traits and unique *repertoire* of bioproducts. However, most of these microbes require modifications to improve their growth and production capacities as well as robustness in terms of genetic stability. For this, genome reduction is a valuable and powerful approach to meet industry requirements and to design highly efficient production strains. Here, we provide an overview of various genome reduction approaches in prokaryotic microorganisms, with a focus on non-model organisms, and highlight the example of a successful genome-reduced model organism chassis. Furthermore, we discuss the advances and challenges of promising non-model microbial chassis.

## 1 Introduction

Global warming is a worldwide threatening issue that requires immediate action on all levels, from personal behaviour to industrial production. Net zero CO_2_ emissions need to be achieved by the middle of this century and must be consequently kept from there on (Rogelj et al., 2015; Chen, 2021). To reach this goal, the transition from a fossil-fuel based economy to a bio-based circular economy is required, and industrial biotechnology represents one of its key enabling technologies. Bio-based processes can replace the fossil fuel by deriving chemical products via fewer conversion steps, application of mild process conditions and by avoiding toxic waste formation that is difficult to recycle or degrade (Hatti-Kaul et al., 2007). Hence, new biorefinery principles must be developed and optimised, especially in terms of efficiency. Up to now, the scale-up remains the major challenge, and related key aspects include bioreactor configuration, feedstock pre-treatment and microbial host choice and design (Navarrete et al., 2020). The identification of the proper host and its development towards a microbial chassis represents a crucial step, as it must adapt the organism to the feedstock and process demands, thereby determining the overall efficiency of the process.

Nowadays, only few microbial model organisms are used in the commercial production of amino acids, enzymes or insulin, for example. Traditional microbes, such as *Escherichia coli*, *Bacillus subtilis*, *Saccharomyces cerevisiae* and *Corynebacterium glutamicum* are well established and consequently more exploited. However, different bioprocesses require different traits, and traditional microbes often lack essential requirements such as: high growth rates, easy handling in diverse bioprocesses, robustness against harsh process conditions, and tolerance towards high substrate and product concentrations (Blombach et al., 2021; Jackson et al., 2021). Therefore, the quest of developing new microbial chassis with better traits is essential to fulfil the needs of a sustainable bio-based industry. Collective progress in synthetic biology, metabolic engineering and genome reduction is an important resource to achieve this goal (Ko et al., 2020; Voigt, 2020; Garner, 2021).

Here, we review the current knowledge on microbial chassis development by multiple aspects including genome reduction, genetic manipulation tools, and biotechnological application, focusing on promising non-traditional prokaryotic organisms.

## 2 Microbial chassis development

**FIGURE 1:**
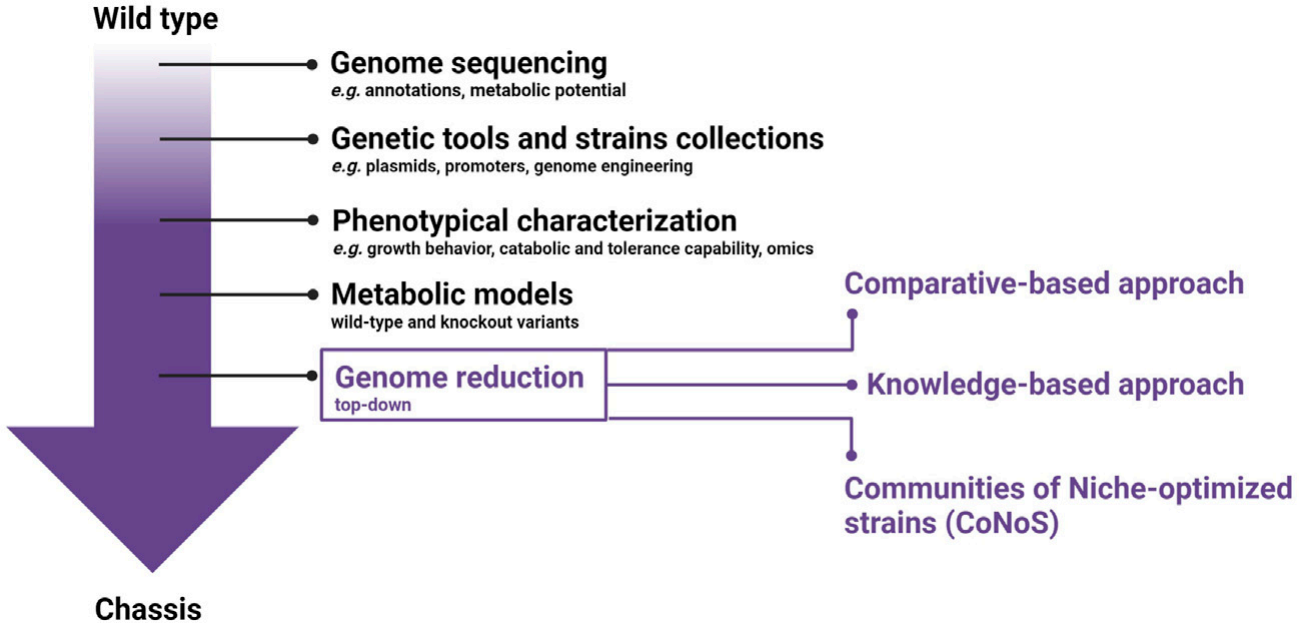
General workflow for chassis development with emphasis on the different approaches available for genome reduction.

A microbial chassis is defined as an “*engineerable and reusable biological platform with a genome encoding several basic functions for stable self-maintenance, growth, and optimal operation but with the tasks and signal processing components growingly edited for strengthening performance under pre-specified environmental conditions”* (de Lorenzo et al., 2021). Briefly, to create a microbial chassis, extensive studies are required to provide a detailed information of the general and metabolic features of the selected strain (Figure 1). To start, the complete genome sequence including comprehensive annotation must be available. The *in silico* analysis of genomic data allows a first evaluation of the metabolic potential. Furthermore, it provides initial knowledge on non-essential genes and putative pathogenic elements that can be targets for the genome reduction strategy. Molecular biological tools, including plasmids and a set of constitutive and inducible promoters of different strengths, have to be developed, alongside genetic engineering tools for precise editing. Ideally, these tools should enable large genomic deletions and multiplexing, enhancing the ease and velocity of genetic manipulations. Omics-based *in silico* technologies built on quantitative physiology can be exploited to guide strain development to achieve high product titers, rates, and yields. Metabolic models are highly needed and must be constructed based on wild-type (wt) genome and data from knockouts variants. The optimization of the microbial chassis should not only aim at improving its bioproduction performance, but should also require an improvement of its robustness towards industrial settings.

## 3 Genome reduction is a valuable approach for achieving robust chassis

The increasingly efficient new-generation sequencing techniques have completely revolutionized biology (Satam et al., 2023). Biological systems are more and more analysed with a holistic genome-based approach instead of single gene analysis, providing a huge amount of data that highlights the complexity of a cell and gives great insights into the metabolism and physiology. In addition, through the increased number of genomes being sequenced, many predicted proteins with no discernible function are identified (Ijaq et al., 2015). Cell complexity causes unpredictable interactions making modelling of the metabolism and functional predictions more challenging (Choe et al., 2016). For this reason, one of the aims of genome reduction is to reduce the complexity of a chassis and hence improve the predictability and controllability. Genome reduction can proceed in either a bottom-up or top-down approach (Figure 1). The bottom-up approach entails designing and building an artificially synthesized genome. Conversely, the top-down approach starts from an intact genome of the target organism and proceeds with a systematic removal of “unnecessary” genes and genomic regions.

For the sake of the simplicity of this review, bottom-up approaches will be discussed in more detail elsewhere (Chi et al., 2019). Yet, it is worth mentioning the significance of the JCVI-syn3.0 project, which successfully created the first minimal cell (Glass et al., 2017). This engineered cell encodes only for 473 genes in its genome, which is a much lower number than any other free-living organism. While this synthetic cell can survive and replicate, the elimination of many non-essential genes caused 3-fold less slower growth rate than that of the parental *Mycoplasma mycoides* and some altered morphological traits. To achieve the goal of an industrial chassis a prior thorough knowledge of gene essentiality is required, especially to maintain proper growth and production. Indeed, insufficient knowledge of essential gene sets, especially of non-model organisms, is the main limitation of the bottom-up approach to directly design an active genome. Based on the enormous progress of AI-based tools for genome editing, metabolic modelling in combination with automation and high-throughput genome editing and screening techniques the progress of genome reduction will be enormously accelerated and increasingly efficient (Zampieri et al., 2019; Li S. et al., 2022; Cheng et al., 2023). These emerging techniques and approaches will be beneficial for both bottom-up as well as top-down approaches, leading to customized and/or new-to-nature strains for specific applications (Kim et al., 2024). Once there will be this extensive progress, there might be a trade-off between choosing which genome reduction approach is more appropriate to use for a certain application. However, for now, the top-down down approach is less costly and relatively straightforward which is why this approach is more widely used.

Nowadays, many prokaryotic strains are subjected to genome reduction, as given in Table 1. Compared with eukaryotic genomes, bacterial genomes are smaller and encode a smaller number of genes. Consequently, genome reduction approaches have been less exploited in eukaryotes and were mainly performed to gain knowledge on cellular function and survival strategies (Kurasawa et al., 2020). The few genome reductions as performed for *S. cerevisiae* and *Saccharomyces pombe* were both valuable from a functional and industrial application perspective (Sasaki et al., 2013; Shao et al., 2018; Postma et al., 2022). An increase in protein production was achieved for *S. pombe* (Sasaki et al., 2013). Although negative characteristics were observed for the genome reduced *S. cerevisiae*, higher levels of ethanol and glycerol were reached (Murakami et al., 2007). A recent approach was used to acquire further knowledge on central carbon metabolism (CCM) by deleting 32 % of CCM-related proteins. No major effects on the growth behaviour were identified. Yet, this study contributes to guide future minimized yeast chassis (Postma et al., 2022).

**TABLE 1:** Genome-reduced strains in prokaryotes.

Parental strain	Genome-reduced strain	Deletion targets	Deletion size	Characteristics	References
*Bacillus amyloquefaciens* LL3	GR167	Genomic islands, extracellular polysaccharide biosynthesis genes, prophages	168 kb (4.2%)	Faster growth, higher transformation efficiency, increased heterologous gene expression	Zhang et al. (2020)
*Bacillus subtilis* 168	Δ6	Prophages, pks operon	323 kb (7.7%)	Normal growth and protein production	Westers et al. (2003)
	MG1M	Prophage regions, antibiotic polyketide genes, sporulation	991 kb (24.0%)	Decreased growth rate, comparable protein production	Ara et al. (2007)
	MGB874	74 genomic regions with prophages, secondary metabolic genes and non-essential genes	814 kb (20.7%)	Decreased growth rate, 1.7-fold increase in cellulase and 2.5-fold protease production, no sporulation	Morimoto et al. (2008)
	BSK814G2	Prophages, antibiotic production genes, nonessential genes	814 kb (20.0%)	Decreased growth characteristics, 4.4-fold higher guanosine production	Li et al. (2016)
	BSK756T3	Prophages, antibiotic production genes, nonessential genes	756 kb (18.6%)	Decreased growth characteristics, 5.2-fold higher thymidine production	
	IIG-Bs27-47-24	Genes for sporulation, motility, secondary metabolism, prophages, pks operon, secreted proteases, and more	1.32 Mb (30.95%)	Close-to-wild-type growth properties, 3000-fold increased secretion of active *Gaussia* luciferase which contains five disulphide bonds and enhanced production of the immunodominant staphylococcal antigen A	Aguilar Suárez et al. (2021), Schilling et al. (2023c)
	PG10	Genes for sporulation, motility, secondary metabolism, prophages, pks operon, secreted proteases, and more	1.46 Mb (34.6%)	Decreased growth rate, reduced flux through glycolysis, altered amino acid metabolism, improved production of difficult to express proteins and lantibiotics	Reuß et al. (2017), Suárez et al. (2019), Van Tilburg et al. (2020)
*Corynebacterium glutamicum* ATCC13032	MB001	3 prophages	205 kb (6.0%)	Improved growth under stress conditions, 30% increased transformation efficiency and plasmid copy number	Baumgart et al. (2013)
	C1*	Irrelevant genes	439 kb (13.4%)	Wild-type-like growth in rich and minimal medium, robust against stresses, improved growth stability	Baumgart et al. (2018)
	CR101	All prophages and IS elements	249 kb (7.6%)	Similar growth rate and transformation efficiency to MB001	Linder et al. (2021)
*Escherichia coli* K-12 MG1655	MDS42	Insertion sequences	663 kb (14.3%)	Normal growth, increased transformation efficiency, and plasmid stability when toxic protein is present	Pósfai et al. (2006)
	Δ16	Nonessential genes	1.38 Mb (29.7%)	Lower growth rate, longer and wider cells, aberrant nucleoid structure	Hashimoto et al. (2005)
	MS56	IS elements, K-islands, flagella, LPS synthesis genes	1.07 Mb (23.0%)	Normal growth aside in M9 basic medium, increased genetic stability, stable plasmid and protein production	Park et al. (2014a)
*Escherichia coli* K-12 W3110	MGF-01	Nonessential genes	1.03 Mb (22.2%)	1.5-fold higher cell density, 2-fold higher threonine production	Mizoguchi et al. (2007)
*Halomonas bluephagenesis* TD01	ΔPS1234Δ50k	Flagella, exopolysaccharides and O-antigene gene clusters	121 kb (3.0%)	Normal growth and PHB production, self-flocculation favouring downstream process, electrotransformation finally feasible	Xu et al. (2022)
*Lactococcus lactis* NZ-9000	9K-4	Prophages, integrases, and transposases	72 kb (2.8%)	Faster growth rate, increased biomass yield, improved heterologous gene expression up to 4-fold	Zhu et al. (2017)
*Lactococcus lactis* N8	N8-8	Prophages and genomic islands	176 kb (6.9%)	Shortened generation time by 17%, normal nisin production	Qiao et al. (2022)
*Magnetospirilium Grywaldense*	ΔTZ-17	Prophages, transposases, nitrogen fixation genes, pks operon	228 kb (5.5%)	Normal growth and magnetosome biosynthesis with improved genomic stability	Zwiener et al. (2021)
*Paenibacillus polymyxa* DSM 365	GR1	8 biosynthetic gene clusters and *sacB*	178 kb (3.0%)	Normal growth and normal producuction of 2,3-butanediol and exopolysaccharides	Ravagnan et al. (2024)
	GR2	2 genomic islands and 6 copies of one IS element	39 kb (0.6%)	Normal growth and normal production of 2,3-butanediol and exopolysaccharides	
*Pseudomonas chlororaphis* GP72	MDS22	22 genomic regions with non-essential genes	686 kb (10.3%)	Some strains show decreased growth and production, others display increased phenazine production	Shen et al. (2017)
*Pseudomonas mendocina* NK-01	NKU421	Genomic island, prophages, hypothetical protein clusters	418 kb (7.7%)	Increased ATP/ADP ratio by 11-fold, Improved mcl-PHA and alginate oligosaccharide production by 114.8% and 27.8%, respectively	Fan et al. (2020)
*Pseudomonas putida* KT2440	KTU-13	13 genomic islands	255 kb (4.1%)	45-fold increase in transformation efficiency, 9.4-fold increase in heterologous protein expression, 39% increase in PHA production	Liang et al. (2020c)
	EM383	Flagella, 4 prophages, transposons, restriction modification endonucleases	266 kb (4.3%)	Increased growth rate and biomass yield in minimal medium, reduced maintenance coefficient, higher plasmid stability, and specific protein productivity, higher AEC, reduced organic acid formation	Martínez-García et al. (2014), Lieder et al. (2015)
*Pseudomonas taiwanensis* VLB120	GRC1	Megaplasmid, 5 prophages, flagella and biofilm genes	640 kb (10.7%)	Increased growth rates and biomass yield, 1.47-fold improved productivity of phenol	Wynands et al. (2019)
*Schlegelella brevitalea* DSM 7029	DC1-7	7 biosynthetic gene clusters	316 kb (4.9%)	Improved or decreased growth, higher transformation efficiency circa 3-fold on average	Liu et al. (2021)
	DT1-10	Transposons, 2 prophages, genomic islands, 3 biosynthetic gene clusters	316 kb (4.9%)	Higher cell growth and biomass, higher transformation efficiency 16-fold on average, shorter and smaller cells, alleviation of autolysis, increased heterologous antibiotic production (e.g., 14-fold higher myxochelin A)	
*Streptomyces albus* J1074	Del14	15 biosynthetic gene clusters	500 kb (7.3%)	Normal growth and improved heterologous gene expression of 7 products by circa 2-fold, simplified metabolic background	Myronovskyi et al. (2018)
*Streptomyces avermitilis* K139	SUKA17	Biosynthetic gene clusters, prophages, transposases	1.67 Mb (18.5%)	Higher cell growth rate and biomass, higher heterologous production (e.g., 4-fold increase streptomycin)	Komatsu et al. (2010), Komatsu et al. (2013)
*Streptomyces coelicolor* M145	M1152 and M1154	4 biosynthetic gene clusters with introduced point mutations in *rpoB* and *rpsL*	173 kb (2.0%)	Comparable growth but not when spores were used as inoculum, 40-fold increased chloramphenicol and circa 30-fold increased congocidine production, simplified metabolic background	Gomez-Escribano and Bibb (2011)
	ZM12	10 biosynthetic gene clusters and a subtelomeric segment	1.22 Mb (14.0%)	Normal growth, some intermediate strains show increased actinorhodin production	Zhou et al. (2012)
*Streptomyces chattanoogenesis* L10	L321	7 biosynthetic gene clusters	736 kb (7.7%)	Normal growth, increased ATP and NADPH availability, higher transformation efficiency, improved heterologous gene expression, and increased genetic stability	Bu et al. (2019)
*Streptomyces lividans* TK24	ΔYA11	11 biosynthetic gene clusters	229 kb (2.7%)	Slightly higher growth rate and biomass in some of the strains, higher heterologous natural products production (e.g., 4-fold higher deoxycinnamycin), simplified metabolic background	Ahmed et al. (2020)
*Streptomyces rimosus* ATCC 10970	Δ145 kb	2 biosynthetic gene clusters and other nonessential genes	145 kb (1.5%)	Normal growth, transcriptome changes, morphological change, 10-fold higher oxytetracycline, activated cryptic gene clusters (longicatenamycins and tyrobetaine-2)	Pšeničnik et al. (2024)
	Δ240 kb	3 biosynthetic gene clusters and other nonessential genes	240 kb (2.5%)	Normal growth, transcriptome changes, 2-fold higher oxytetracycline, activated cryptic gene clusters (longicatenamycins)	
*Synechococcus elongatus* UTEX 2973	SG33	Nonessential genes	34 kb (1.2%)	23% increased growth rate	Sengupta et al. (2024)
	SG55	Nonessential genes	55 kb (2.0%)	9% increased growth rate, 10-fold higher transformation efficiency, 22.7% sucrose production, no difference in quantum yield compared to the wt	
*Vibrio natriegens* 14,048	Δvnp12	All prophages	75 kb (2.3%)	Normal growth, improved robustness in DNA-damaging and hypo-osmotic conditions, increased pyruvate production 40-fold	Pfeifer et al. (2019)

Concerning prokaryotes, many studies on genome reduction have shown the great benefits of top-down approaches when developing a microbial cell factory: enhancement of genomic stability, higher transformation efficiency, optimization of downstream application and improvement of growth rate and/or bioproduction. The enhancement of genomic stability is reached through the deletion of genetic mobile elements, such as prophages or insertion sequences (IS), and SOS response mechanisms due to causing negative random mutations and/or product inactivation (Csörgo et al., 2012; Park M. K. et al., 2014; Choi et al., 2015; Peng and Liang, 2020; Zwiener et al., 2021). Park et al. developed an IS-free *E. coli* strain, and enhanced the production of two recombinant proteins, the tumour necrosis factor-related apoptosis-inducing ligand (TRAIL) and the bone morphogenic protein-2 (BMBP2) by 25 % and 20 %, respectively (Park M. K. et al., 2014). The deletion of the error-prone DNA polymerases in *E. coli* also showed increased genomic stability and a 50 % decrease in spontaneous mutation rate (Csörgo et al., 2012). To improve downstream applications, the deletion of undesired products is essential. Many studies have focused on the deletion of antibiotics encoding genes, but also on increasing the precursor supply necessary for a target product (Gomez-Escribano and Bibb, 2011; Myronovskyi et al., 2018; Ahmed et al., 2020). Myronovskyi et al. developed a *Streptomyces albus* strain in which 15 native antibiotic gene clusters were deleted. The mutant displayed higher production of 5 heterologous expressed biosynthetic gene clusters (BGCs) of around 2-fold compared to the parent strain (Myronovskyi et al., 2018). Another example is *Streptomyces lividans* in which the deletion of 10 endogenous antibiotic encoding clusters resulted in a higher growth rate and a 4.5-fold increase of the production of the heterologous expressed deoxycinnamycin (Ahmed et al., 2020). Furthermore, in both studies, the metabolic background was greatly simplified for the analysis of secondary metabolite gene cluster expression. Finally, the deletion of unnecessary proportions of a genome at a large scale might lower the operating costs of the living cells (reduction of DNA, RNA and protein synthesis costs) yielding improved cellular performances in terms of growth rates, cell density, and/or productivity compared with their wild-type counterparts (Westers et al., 2003; Mizoguchi et al., 2007; Lieder et al., 2015; Li et al., 2016; Bu et al., 2019; Wynands et al., 2019; Liang et al., 2020b; Fan et al., 2020; Zhang et al., 2020; Qiao et al., 2022; Pšeničnik et al., 2024; Sengupta et al., 2024). A 2.83 % genome-reduced *Lactococcus lactis* strain showed faster growth, increased biomass yield, and improved protein production of up to 4-fold (Zhu et al., 2017). Also, in another study different genome-reduced strains of *Pseudomonas putida* were developed reaching a total of 4.3% genomic deletion. They were subsequently tested also in bioreactors and proved to be more efficient than the wild type in growth rate, biomass yield, plasmid stability, and viability (Martínez-García et al., 2014; Lieder et al., 2015).

When aiming to reduce a genome, the major challenge is the choice of the targets for deletions. So far, mainly two strategies are applied. The first approach uses comparative genomics to identify essential genes to avoid the deletion of indispensable functions (Kolisnychenko et al., 2002; Suzuki et al., 2005b; Liang et al., 2020b). The second approach is more knowledge-based and aims at deleting prophage regions, insertion elements, and secondary metabolites (Li et al., 2016; Baumgart et al., 2018; Myronovskyi et al., 2018; Ahmed et al., 2020; Ravagnan et al., 2024). However, undesired side effects can still be observed (Hashimoto et al., 2005; Murakami et al., 2007; Reuß et al., 2017; Shen et al., 2017; Qiao et al., 2022). Noack and Baumgart systematically categorized the whole genome of *C. glutamicum* in relevant, non-essential and irrelevant genes. Relevant genes are those which are necessary for survival, while non-essential and irrelevant ones are the usual targets for genome reduction (Noack and Baumgart, 2019). Mainly, non-essential genes are a subset of relevant genes that have no effect on cell viability but can be beneficial for cell growth in certain conditions. Instead, irrelevant genes are highly expressed genes whose products are not required under the defined cultivation conditions and they represent the most promising target genes for deletion. The genome-reduced strain can still replicate on its own while displaying advantageous production traits in the defined environment. To define this set of genes, transcriptomics and proteomics data should be included in the desired environment which should mimic as closely as possible the industrial conditions. This approach should be considered and performed for each target product, since different operating conditions may be required. Another promising concept for genome reduction is the Community of Niche-optimized strains (CoNoS), where two strains of the same species are each carrying one or more auxotrophies and cross-feed each other, saving carbon and energy (Noack and Baumgart, 2019). This strategy was successfully applied for the first time in 2022 using two amino acid auxotrophic *C. glutamicum* strains (Schito et al., 2022). Synthetic communities can be established between genome-reduced strains growing with mutual dependency and opening up to new future perspectives.

## 4 Microbial chassis from a historical example to new promises

Decades of technological developments have established *E. coli*, *S. cerevisiae*, *B. subtilis,* and *C. glutamicum* as important workhorses among others. Indeed, these traditional organisms have advantages as chassis cells due to the manifold efficient synthetic biology tools for engineering and well-analyzed genomes and metabolic networks. However, the growing number of more and more versatile and sophisticated bioprocesses and bioproducts bring these established workhorses to their limit from a metabolic point of view. Consequently, new microbes are emerging and gaining more relevance as future chassis. In the section below, we present *C. glutamicum* as a great example of a model platform chassis, with a special focus on its industrial relevance. Following, we will present an overview of alternative promising microbes with great potential for the production of different value-added products based on their specific characteristics (Table 2).

**TABLE 2:** Characteristics of microbial chassis.

Chassis cell	Characteristics				
	Products	Highest genome reduction achieved (%)	Benefits	Open needs	References
*Corynebacterium glutamicum*	L-amino acids (*e.g*., glutamate and lysine), organic acids, diamides and more	13.4	Assimilation of carbon from a variety of carbon sources, simultaneous utilization of a mixture of carbon sources, tolerance towards organic acids, robust, high cell densities, optimised production strains, genome wide metabolic models, tools for genome engineering and gene expression	Multiplex genome editing tools, more efficient plasmid curing systems	Baumgart et al. (2013), Baumgart et al. (2018), Baritugo et al. (2018), Wolf et al. (2021), Kim et al. (2023)
*Halomonas bluephagenesis*	PHAs, ectoine	3.0	Contamination resistant, high cell densities, tools for genome engineering and gene expression	More versatile genome editing tools compared to traditional microbes	Zhao et al. (2017), Qin et al. (2018), Shen et al. (2018), Ma et al. (2020), Ma et al. (2022), Ye et al. (2020), Xu et al. (2022)
*Paenibacillus polymyxa*	*R,R*-2,3-butanediol, exopolysaccharides, antibiotics (e.g., polymyxin)	3.0	Assimilation of carbon from a variety of carbon sources, versatile metabolism and tools for genome engineering and gene expression	More versatile genome editing tools compared to traditional microbes	Häßler et al. (2012), Grady et al. (2016), Brito et al. (2017), Rütering et al. (2017), Schilling et al. (2021), Meliawati et al. (2022), Sun et al. (2022), Ravagnan et al. (2024)
*Schlegelella brevitalea*	Glidobactins and heterologous production of other NRP/PKS (e.g., epothilones)	4.9	Tools for genome engineering and gene expression, outstanding biosynthetic capacity for dozens of secondary metabolites	Early autolysis, more versatile genome editing tools compared to traditional microbes	Wang et al. (2018b), Yu et al. (2020b), Wang et al. (2021b), Liu et al. (2021)

### 4.1 *Corynebacterium glutamicum*

*Corynebacterium glutamicum* is a facultative anaerobic bacterium that has been used safely for more than 50 years in the food and feed industry. It is commercially used for the production of L-amino acids including the dominating products L-glutamate and L-lysine (Park S. H. et al., 2014; Bampidis et al., 2019). It is also an efficient platform organism for the biotechnological production of diverse and valuable products like organic acids, carotenoids, fuels, diamines, and more (Becker et al., 2018; Wolf et al., 2021).

*C. glutamicum* represents an excellent workhorse for several reasons. *C. glutamicum* can assimilate a variety of different carbon sources, like sugars, organic acids, and aromatic compounds simultaneously which renders it superior compared to *E. coli*, *B. subtilis* or *S. cerevisiae* (Baritugo et al., 2018; Inui and Toyoda, 2020). In addition, *C. glutamicum* shows only a weak carbon catabolite inhibitory effect on mixed carbon sources, which is highly beneficial for industrial applications (Wendisch et al., 2000; Buschke et al., 2013). However, it cannot directly utilize complex polysaccharides, such as xylan, cellulose or starch (Baritugo et al., 2018). For this reason, it has been engineered for consolidated bioprocessing by the heterologous expression of amylolytic and cellulolytic enzymes to produce different biochemicals (Lee et al., 2014; Yim et al., 2017). Another important feature is its robustness and tolerance to organic acids, furfural, and toxic aromatic compounds as obtained from hydrolysis of next-generation feedstocks such as lignocellulosic biomass. Moreover, *C. glutamicum* cells maintain a strong catalytic function and high-density fermentation under growth-inhibiting conditions (Chen et al., 2017; Conrady et al., 2019; Kitade et al., 2020).

Genomic robust strains, which are free from the adverse effects of prophages and IS elements, are highly demanded in industrial settings. *C. glutamicum* is a great example of how the deletion of such destabilizing genomic elements can improve bioproduction. Choi et al. deleted the major IS elements and used the resulting mutant for the plasmid-borne production of poly(3-hydroxybutyrate) and γ-aminobutyrate. The authors observed an improved productivity of both products (Choi et al., 2015). The first genome-reduced *C. glutamicum* (MB001) targeted the three prophage sequences of its genome, resulting in a total genome reduction of 6 %. Under SOS-response conditions, in which one of the bacteriophages is induced, the prophage-free variant showed improved growth, higher transformation efficiency, and 30 % increased production of the enhanced yellow fluorescent protein (eYFP) compared to the wild type (Baumgart et al., 2013). The MB001 strain was further engineered by deleting all copies of two IS elements and was used as a starting point for the singular deletion of 36 predicted nonessential regions. 26 of these regions proved not to affect biological fitness (Unthan et al., 2015). Combinatorial deletions of these irrelevant gene clusters resulted in a library of 28 strains that were characterized by different stress conditions. The final *C. glutamicum* C1* with a total reduced genome of 13.4 % represents an ideal base for further improvements toward a biotechnologically relevant chassis (Baumgart et al., 2018). Many of these genome-reduced mutants were used for the production of various value-added compounds, as reported (Lara and Gossett, 2019). Recently, Linder et al. have deleted all the IS elements of *C. glutamicum* starting from the MB001 strain (Linder et al., 2021). Additionally, recent studies have also demonstrated their further industrial potential (Henke and Wendisch, 2019; Haupka et al., 2020; Hemmerich et al., 2020; Walter et al., 2020; Banerjee et al., 2021; Gauttam et al., 2021; Ferrer et al., 2022; Kiefer et al., 2022; Mindt et al., 2022; Prell et al., 2022; Schmitt et al., 2023). For example, 3-hydroxycadaverine was produced *de novo* by the lysine overproducing genome-reduced variant, resulting in the highest titer of this compound reported till now of 8.6 g/L (Prell et al., 2022). Mindt et al. produced indole by exploiting the C1* genome-reduced chassis due to its higher product tolerance and lack of a flavin-dependent monooxygenase which is known to oxidize indole to indoxyl (Mindt et al., 2022; Mindt et al., 2023).

A commonly used approach for editing the genome of *C. glutamicum* is based on the use of a two-step recombination system with a conditionally lethal marker, such as the levansucrase encoding gene *sacB* (Niebisch and Bott, 2001). Other genetic tools described are the Cre/loxP system and the RecT-mediated single-stranded recombination (Suzuki et al., 2005a; Huang et al., 2017). These procedures are often laborious and/or lack the simultaneous editing of multiple genes. Recent studies have developed two different CRISPR-Cas systems for genome editing: one utilizing Cfp1 (Cas12a) from *Francisella novicida*, and the other utilizing Cas9 from *Streptococcus pyogens* (Jiang et al., 2017; Liu et al., 2017). Both approaches resulted in large nucleotide sequences deletions and integrations and single-nucleotide exchanges were realized. Jiang et al. also achieved double-locus editing (Liu et al., 2017). However, this system still requires optimization concerning editing efficiencies. To improve the homologous repair activity CRISPR systems combined with the recombinase RecT system were successfully established (Cho et al., 2017; Wang B. et al., 2018; Li et al., 2021). The recombineering efficiency reached the highest efficiency reported of 13,250 transformed cells/10^9^ viable cells (Li et al., 2021). The CRISPR interference (CRISPRi) technology for gene repression has been widely used with the nuclease deactivated dCas9 and dCfp1 (Cleto et al., 2016; Zhang et al., 2016; Liu et al., 2019; Li et al., 2020; Göttl et al., 2021; Li et al., 2022a). Other synthetic biology tools for controlling gene expression through static and dynamic regulation have also been investigated in *C. glutamicum* (Yim et al., 2013; Shi et al., 2020). Many synthetic promoter libraries were also generated (Zhang et al., 2015; 2018; Duan et al., 2021). To regulate the metabolic flux in real-time, dynamic regulatory systems were employed (Tan et al., 2020; Ferrer et al., 2022; Lai et al., 2022). Finally, *C. glutamicum* counts different genome-metabolic models developed to guide metabolic engineering targets by improving experimental design (Kjeldsen and Nielsen, 2009; Mei et al., 2016; Feierabend et al., 2021; Niu et al., 2022; Zhang et al., 2022). Deeper insights into available synthetic tools in *C. glutamicum* are further reviewed elsewhere (Kim et al., 2023).

*C. glutamicum* has emerged as a potent host to produce molecules for human health and wellbeing and has greatly expanded its role from a traditional producer of amino acids to a multi-functional microbial production platform. Its valuable native and engineered traits secured its establishment in industry and drive the rapid and continuous technical improvements that help gain deeper insights into *C. glutamicum* physiology and genetics.

### 4.2 *Halomonas bluephagenesis*

*Halomonas bluephagenesis* is an auto sterile halophilic bacterium, with high potential for next-generation industrial biotechnology (NGIB) (Chen and Jiang, 2018). Due to its fast growth in high salt and alkaline conditions, open and continuous processes were conducted for 2 months without any contamination (Tan et al., 2011). Successful cases of unsterile pilot-scale productions were also performed with significant cost reductions, assessing *H. bluephagenesis* as a suitable industrial strain for large-scale production under open non-sterile conditions (Ye et al., 2018). To support its growth in such unusual environments, it produces different compounds, such as the compatible solute ectoine and polyhydroxyalkanoates (PHAs), such as polyhydroxybutyrate (PHB) which is naturally produced as an energy and carbon storage and stress resistance enhancer, up to 80 % of cell dry mass (Tan et al., 2011; Obruca et al., 2018).

PHAs are a family of biodegradable and biocompatible biopolymers with the potential to replace petroleum-based plastics (Keshavarz and Roy, 2010). They can be used in various fields ranging from packaging to biomedical applications (Valappil et al., 2006; Rehm, 2010; Możejko-Ciesielska and Kiewisz, 2016). Since new PHAs are industrially required to provide different material properties that can suit more applications, *H. bluephagenesis* has also been genetically engineered to produce PHBV, P3HB4HB, PHA copolymyers P(3HB-co-3HHx), and many others (Yu L. P. et al., 2020; Mitra et al., 2020; Yan et al., 2022). To further increase PHA accumulation, many efforts have been made to improve the competitiveness of microbial production via synthetic biology and metabolic engineering.

*H. bluephagenesis* has been manipulated mainly via a markerless gene replacement procedure mediated by double-strand breaks and via a CRISPR-Cas9 based system (Fu et al., 2014; Qin et al., 2018; Liu et al., 2023). The latter was used to engineer the TCA cycle for PHBV production and for *de novo* synthesis of the 4-hydroxybutyrate-CoA (4HB) pathway for P3HB4HB production (Chen et al., 2019; Ye et al., 2020). Further optimization approaches for PHA production include the investigation of a set of different promoters, starting from P_porin_ which is a strong constitutive promoter commonly used in *Halomonas* sp. Based on mutations in the core sequence of this promoter, a promoter library with a wide range of transcriptional strength was created and its tunability was then demonstrated by regulating the PHA biosynthetic pathway (Shen et al., 2018). A novel type of T7-like inducible promoter systems were identified, one of which was used to express the cell-elongation cassette and PHB biosynthetic pathway reaching a 100-fold increase in cell lengths and a final concentration of 92 % PHB by cell dry weight which is the highest titer reached so far (Zhao et al., 2017). By the use of high-resolution control of gene expressions (HRCGE) system, the 4HB pathway was fine-tuned by titrated IPTG concentrations and varying promoter combinations. Once the fine-tuned pathway was integrated into the genome of the mutant of the two succinate-semialdehyde dehydrogenases (Δ*gabD2* and Δ*gabD3)*, P3HB4HB synthesis was realised and reached 75 % (wt) (Ye et al., 2020). Other studies designed a synthetic oleic acid induced promoter and two other inducible systems induced by acyl homoserine lactone and IPTG for controllable gene expression (Ma et al., 2020; Ma et al., 2022). A CRISPR interference approach was also established in *H. bluephagenesis*. This system was used to repress the 2-methylcitrate synthase *prpC* and the citrate synthase *gltA* genes which reached the accumulation of PHBV of 82 % and 72 % (wt), respectively, without affecting growth (Tao et al., 2017). Recently, the CRISPR-Cas9 genomic engineering system developed by Qi et al. was further optimized by using dual-sgRNA enabling large genomic deletions up to 50 kb. In sum, 3 % of the whole genome was reduced by deleting flagella, exopolysaccharides (EPSs), and O-antigen gene clusters. For the first time, this mutant was efficiently transformed through electroporation and gained the ability to self-flocculate which facilitates separation procedures. This achievement is the first genome-reduced *Halomonas* strain (Qin et al., 2018; Xu et al., 2022).

Although *H. bluephagenesis* synthesize only PHB from unrelated carbon sources, it has emerged as a strong candidate for industrial PHA production with the establishment of a tractable genetic system, the completion of genome sequencing, and the wide application of metabolic engineering. Finally, by producing ectoine, this microbe stands also as a future chassis for the co-production of different valuable biomolecules in open unsterile, and continuous growth conditions (Ma et al., 2020; Hu et al., 2024).

### 4.3 *Paenibacillus polymyxa*

*Paenibacillus polymyxa* is a facultative anaerobe and endospore forming bacterium which is used as a biofertilizer due to its plant growth-promoting abilities: it solubilises phosphate, fixates nitrogen and acts as a biocontrol agent against different pathogens. As such, *P. polymyxa* produces a wide range of antibiotic compounds which are of great interest in agriculture, medicine and food processing (Padda et al., 2017; Jeong et al., 2019; Langendries and Goormachtig, 2021). Furthermore, it has garnered an enormous interest for its ability to use different carbon sources and for the production of *R,R*-2,3-butanediol (2,3-BDL), and EPS (Grady et al., 2016; Schilling et al., 2020).

2,3-BDL and its derivatives are used in a variety of applications, from next-generation fuels, precursor for pharmaceuticals, cosmetics or food preservatives. Nowadays, there is a strong need of an optimised environmentally friendly alternative (Maina et al., 2022). By mainly producing the relevant (2*R*,3*R*) isomer, *P. polymyxa* DSM 365 is regarded as an efficient producer ensuring a great advantage with a higher purity compared to other known pathogenic 2,3-BDL producing organisms. Physical parameters and fermentation conditions have a profound effect on the final product concentration, as shown in many studies (Häßler et al., 2012; Okonkwo et al., 2017; Ju et al., 2023). With 111 g/L, Häßler et al. have reported the highest concentration obtained by *P. polymyxa* to date (Häßler et al., 2012). Improvements of the 2,3-BDL production were also achieved through genetic engineering. For the first time, Schilling et al. rationally metabolic engineered *P. polymyxa* improving the carbon flux towards 2,3-BDL by deletion of one lactate dehydrogenase and overexpressing the butanediol dehydrogenase (Schilling et al., 2020). The same authors were also able to increase the production by using the full potential of a CRISPR mediated transcriptional perturbation tool in which three *ldh* genes were downregulated at the same time with the upregulation of the *bdh* gene and showed the redirection of the carbon flux from lactate to 2,3-BDL (Schilling et al., 2021).

Within the last few years, *P. polymyxa* has gained more interest also as a producer of different interesting exopolysaccharides (Raza et al., 2011; Rütering et al., 2016). They are used in a variety of applications from food to cosmetics to pharmaceuticals and many others (Freitas et al., 2015; Paul et al., 1986; Schilling et al., 2020). *P. polymyxa* is a natural and avid producer of exopolysaccharides which showed to have great potential new properties compared to already commercialized ones (Rütering et al., 2017, Rütering et al., 2018). Depending on the carbon source and the C/N ratio, it can produce a levan-type polyfructan or heteropolysaccharides (Rütering et al., 2016). After many attempts over the last 50 years, using combinatorial knock-outs of functional genes and by combining analytical methods, it was finally discovered that the heteroexopolysaccharide produced by *P. polymyxa* consists of three distinct polymers which were named paenan I, paenan II and paenan III (Schilling et al., 2022; Schilling et al., 2023b).

Many of these discoveries were facilitated by the efficient editing system based on CRISPR-Cas9 developed in 2017 (Rütering et al., 2017). This system was successfully applied further for large genomic deletions up to 59 kb using one single sgRNA and multiplexing modifications (Rütering et al., 2017; Meliawati et al., 2022; Ravagnan et al., 2024). Indeed, this tool enabled the construction of two robust genome-reduced variants lacking the production of endogenous biosynthetic gene clusters or nonessential genomic regions including the insertion sequence IS*Pap1*. The growth characteristics and the desired product formation of the two variants were maintained at wt-level, an extremely important achievement and starting point for future targeted genetic modifications (Ravagnan et al., 2024). To add to the list of molecular biology tools available in this species, a multiplex base editing system by fusing the dCas9 with a cytidine deaminase to mediate C to T substitutions and, as previously mentioned, a CRISPR-Cas12a tool for mediated activation (CRISPRa) and interference (CRISPRi) were developed (Kim et al., 2021; Schilling et al., 2021). The latter tool enabled a deeper understanding of *P. polymyxa* metabolism both for 2,3-BDL and EPS production and could help guide other future metabolic engineering strategies (Schilling et al., 2021). Aside from CRISPR systems, other genetic engineering tools have been described, even if less efficient (Choi et al., 2008; Kim and Timmusk, 2013). Finally, heterogeneous promoters and inducible promoters have already been tested in *P. polymyxa* (Brito et al., 2017; Wang et al., 2022; Wang et al., 2024). Heinze et al. evaluated 11 different promoters from *Bacillus* species for the expression of cellulases in *P. polymyxa* (Heinze et al., 2018). Through high-throughput screening, a novel strong and constitutive promoter P_*LH-77*_ was identified (Li et al., 2019). The same authors investigated 22 native promoters based on a transcriptomic dataset in which they identified another very strong promoter, especially for the optimization of indol acetic acid (IAA) production (Sun et al., 2022).

As a non-pathogen, *P. polymyxa* has great potential as a cell factory especially due to its versatile metabolism and the available tools for both genetic and metabolic engineering. The recent tool developed by Meliawati et al. substantially advanced the application of the Cas9-based approach for *P. polymyxa* (Meliawati et al., 2022). Additionally, the discovery made by Schilling et al. increased the knowledge of this versatile producer paving the way for new approaches toward tailor-made exopolysaccharides with desirable material properties for medical and industrial applications that could outcompete existing oil-based polymers (Schilling et al., 2022; Schilling et al., 2023a; Schilling et al., 2023b).

### 4.4 *Schlegelella brevitalea*

The reputation of *Schlegelella brevitalea* is mostly due to its native production of the novel type of antifungal and antitumor glidobactins, and its efficient heterologous production of several kinds of secondary metabolites, especially the antitumor epothilones (Bian et al., 2014; Bian et al., 2017; Wang X. et al., 2018). Currently, the original producer *Sorangium cellulosum* is used in the industrial production of epothilones (Gerth et al., 1996). However, this strain shows many disadvantages, such as slow growth and difficulties in genetic modifications (Julien and Shah, 2002; Julien and Fehd, 2003). Therefore, much work has been done to improve epothilones production by *S. brevitalea*. The epothilone cluster of *S. cellulosom* was integrated into the genome of *S. brevitalea* DSM 7029 resulting in the 75-fold increased epothilone yield of 307 μg/L through the optimization of the medium and introducing propionyl-CoA carboxylase (PCC) genes and rare tRNA genes (Bian et al., 2017). Reintegration of another epothilone cluster from a different strain of *S. cellulosum* and again engineering of the precursor pathway enabled an improved titer of 82 mg/L in 6-day fermentation, a comparable result to the industrial producer *S. cellulosum* (Yu Y. et al., 2020). Further studies focused on other approaches to improve its production, starting from transporter engineering, overexpression of ferredoxin genes, functional hybrid polyketide synthases (PKS) generation, swapping domains, and both random and site-directed mutagenesis (Liu et al., 2018; Liang J. et al., 2020; Wang H. et al., 2021; Wang et al., 2023).

A series of genome-reduced *S. brevitalea* chassis strains were developed and tested for its heterologous BGC expression capacity (Liu et al., 2021). Two parallel deletion routes were taken: the first one deleted 8 BGCs (DC mutants), the second one sequentially removed few BGCs, several transposases, GIs, prophages, and vicinal nonessential genes (DT mutants). The DT mutants showed decreased autolysis with related improved growth characteristics, 16-fold higher transformation efficiencies, and improved yields of heterologous secondary metabolites up to 13-fold. These results demonstrate the feasibility and improved robustness of these genome-reduced mutants for secondary metabolite expression compared to both the wild type and common platform chassis *E. coli* and *P. putida*.

With an efficient genome editing system based on the novel bacteriophage recombinases Redαβ7029, which was recently further improved for multiplexing (Wang X. et al., 2018; Wang X. et al., 2021), several native promoters were tested for heterologous expression (Ouyang et al., 2020; Liu et al., 2021) and successful production of different heterologous secondary metabolites including epothilones, vioprolides, rhizomides, holrhizin, and more (Yan et al., 2018; Zhong et al., 2021), *S. brevitalea* stands out as promising future chassis.

## 5 Conclusions and future perspectives

Throughout the years industrial biotechnology has made remarkable progress. Traditional microbes are continuously genetically improved and new microbes are identified and brought towards development as future chassis. Unconventional microbes possess unique characteristics and consequently are required for more tailored bioprocesses to cover the whole industrial product portfolio and the whole substrate field. Still, there is a need to expand the synthetic biology, systems biology and high-throughput techniques to a wider range of bacteria, especially these non-model organisms. Gathering more and more data enables the creation of new available tools for the specific microbe, and data-driven gene annotations advance these less studied organisms to “model-organism” status. Accurate gene annotations would also favour the construction of more reliable, robust, genome-reduced strains. Once many well-established genome-reduced strains will be created, they should be rendered available to the community to use them as starting strains to further engineer and exploit in industry. This might be done by providing them in publicly available strain collections such as ATTC, DSMZ or Addgene, or providing them in an academic background similar to the Keio collection of *E. coli* of which several millions of samples have been distributed by the National Institute of Genetics of Japan. This will massively help to speed up the development of efficient production strains in the future.

In this review, we have delved into the benefits provided by genome reduction strategies and their future directions, also by describing how genome reduction has been applied in *C. glutamicum* achieving an efficient chassis. As we have also reported, genome reduction has started to be applied in non-model organisms demonstrating its relevance and feasibility as a tool for host optimisation. Indeed, we envision new genome-reduced strains with improved characteristics soon.
